# Traumatic Death due to Simultaneous Double Spine Fractures in Patient with Ankylosing Spondylitis

**DOI:** 10.1155/2015/590935

**Published:** 2015-09-08

**Authors:** Mitsuru Yagi, Shunsuke Sato, Atsushi Miyake, Takashi Asazuma

**Affiliations:** Department of Orthopedic Surgery, National Hospital Organization Murayama Medical Center, Japan

## Abstract

The aim of this study is to report the rare occurrence of simultaneous double spine fractures in a patient with progressive ankylosing spondylitis (AS). An 82-year-old male with established AS had low-energy falls. He had sustained simultaneous double spine fractures and died. Plain radiographs of the cervical spine were unremarkable in detecting a cervical spine fracture in a patient with AS and a spinal cord injury following a fall. CT scan showed a displaced fracture at the C6/C7 with American Spinal Injury Association-A spinal cord injury and displaced fracture at L1. The cause of death was determined to be upper spinal cord injury caused by cervical spinal fracture and dislocation that were facilitated by spinal rigidity from AS. This case report illustrates the importance of obtaining a detailed medical history and thorough imaging study when investigating deaths, including nonfatal conditions, such as AS. Furthermore, it shows the value of entire spine CT scan in the evaluation of the mechanism, further spine fractures, and manner of death. Despite the occurrence of spine fracture in AS patients, simultaneous double or multiple spine fractures are extremely rare and can be missed. Care should be taken for the further spine fracture in the entire spine in patient with AS.

## 1. Introduction

The characteristic pathological regions of ankylosing spondylitis (AS) are an ossification of the spinal discs, joints, and ligaments, leading to progressive rigidity and altered biomechanical properties of the spine [1, 2]. The estimated incidence of AS in the general population is around 1% [3, 4]. Since the first reported case of cervical spinal cord injury (SCI) associated with AS in 1933, several similar reports have appeared only sporadically [5–15]. In patients with AS, the occurrence of traumatic cervical spine injury is 3 to 5 times greater than the incidence in the normal population [16–18]. In several large series of cases with SCI, the percentage of patients with associated AS has varied between 0.3% and 0.5% [3, 4, 19]. The rigidly fused spine, named “bamboo spine,” is subsequently extremely unstable with an associated high risk of neurological deterioration. The reported prevalence of clinical vertebral fractures varies widely (approximately between 10% and 20%) [8–10] and the incidence of major neurologic complications following a vertebral fracture in AS is high due to sustaining displaced spinal fractures (29%–91%) [3, 4, 6, 11, 14]. Disproportionately higher morbidity and mortality rates are also reported in the SCI patients with AS [1, 4, 5, 11–13]. The consequence of progressive kyphosis, impaired balance musculoskeletal weakness, and osteoporosis reduces the ability to take protective measures during a fall and can be the risk of spine fractures. The development of a fracture can occur with even a minor trauma [1, 3, 4, 20]. Because of the abnormalities of the spine and affected site of fracture associated with AS (e.g., kyphosis, fusion, osteoporosis, and mostly affected in lower cervical spine), spine fracture can be missed [2, 21]. Among them, simultaneous multiple spine fractures are extremely rare. While remote spine fracture or multiple spine fracture is sometimes seen in patient with high energy trauma, it never occurs by a minor trauma in a healthy population. To the best of our knowledge, simultaneous multiple spine fractures in a single patient with AS have been quite rarely addressed in the literature. Only 2 reports have existed. Osgood et al. have reported multiple spine fracture occurrence at cervical spine and thoracic spine in 1975 [10]. Samartzis et al. have reported multiple fracture occurrence and treatment in the postoperative AS spine with preexisting internal instrumentation for previous fracture treatment in 2010 [22]. Our report is a case of elderly patient with AS who had simultaneously double spine dislocation fractures at cervical spine and lumbar spine and subsequent death by pulmonary failure.

## 2. Case Report

**Figure 1:**
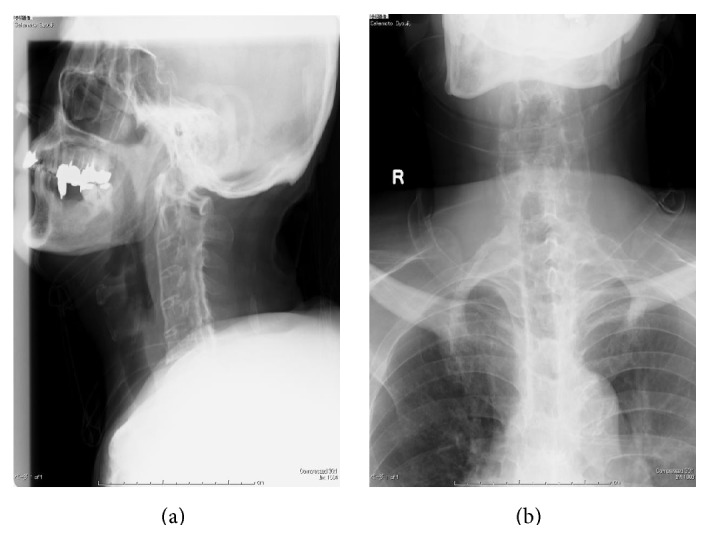
Plain radiographs of the cervical spine were unremarkable in detecting a cervical spine fracture in a patient with AS although the typical radiographic features of AS were apparent in entire spine ((a) AP and (b) lateral).

**Figure 2:**
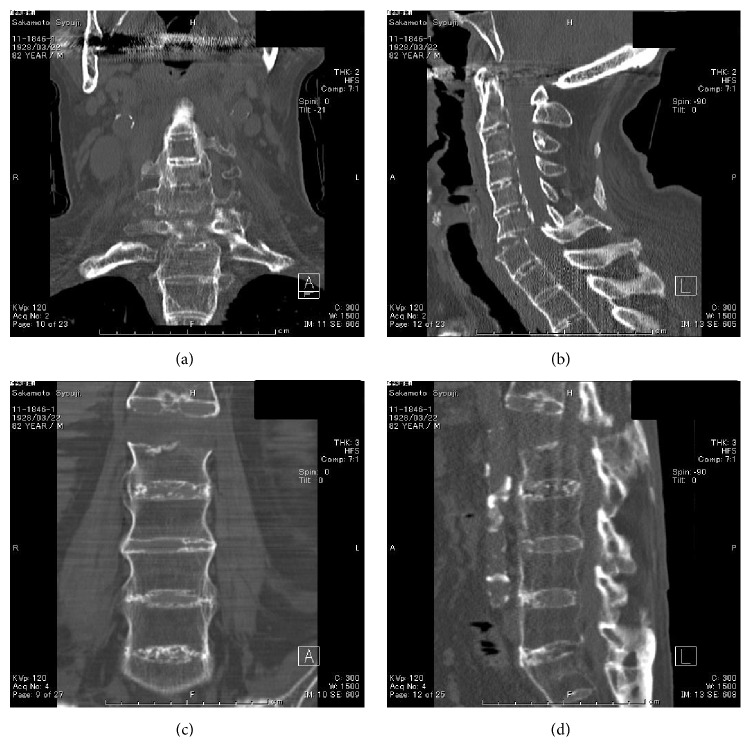
CT scan shows simultaneous cervical and lumbar spine fractures and dislocations at the level of C6-C7 ((a) and (b)) and L1 ((c) and (d)).

**Figure 3:**
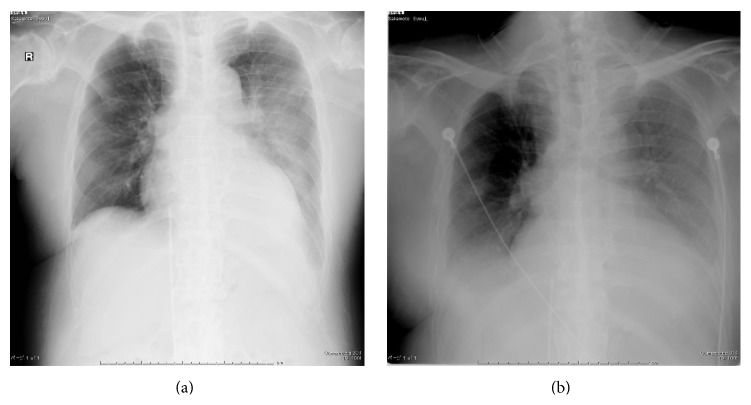
Progressive respiratory failure of the patient ((a) 1 hour after injury and (b) 18 hours after injury).

The informed consent has been obtained from the patient or appropriate persons for publication, including any necessary photographs. Eighty-two-year-old Asian male with a known history of AS and a history of diabetes mellitus and hypertension slipped and fell in the bathtub striking his head and neck. Immediately after the fall, he experienced bilateral motor loss from the C6 level distally, as well as sensory loss. Plain radiographs of the cervical, thoracic spine were unremarkable in detecting a cervical spine fracture in a patient with AS and a SCI following a fall although the typical radiographic features of AS were apparent in entire spine (Figure 1). The patient was placed in a halo vest orthosis. Following halo placement, a high resolution CT scan with sagittal reconstructed views of the entire spine was performed in an attempt to visualize possible cervical spine fracture or dislocation because of the obvious American Spinal Injury Association- (ASIA-) A spinal cord injury at C6/C7 level. A CT scan with sagittal reformatting of the cervical, thoracic, and lumbar region was performed and revealed a displaced fracture at the C6/C7 level as well as fracture and dislocation of the spine at L1 level (Figure 2). Although he was treated with an aggressive medical support, he died of respiratory failure 20 hours after initial injury (Figure 3).

## 3. Discussion

Elderly patients, especially those with preexisting conditions, have increased injury severity compared with younger patients, despite lower levels of traumatic impact [19, 23]. In addition, elderly patients have increased morbidity, mortality, and lower functional status compared with younger cohorts with equal or greater injury severity [19, 23]. The management of cervical fractures in AS reveals a high rate of spinal cord injuries and complications, including a high mortality rate [1, 3, 4, 6, 11, 14]. The lower cervical spine is the most commonly injured region of the spine, followed by the thoracolumbar junction [5, 6, 11, 14]. Fractures tend to occur at the level of the intervertebral disc as a result of incomplete ossification of the nucleus pulposus [22]. Most neurologic injuries in this setting occur at the time of injury, or before stabilization in traction or a halo vest [1, 5, 6, 11, 14]. Consistent with previous 2 reports, age of the patient in this report is high and prognosis is devastating (Table 1). The patient had ASIA Grade A SCI and died of respiratory failure after injury. This result strongly suggests that, even in the minor accident, elderly patient with AS can injure severe multiple spine fractures and can show severe life-threatening SCI due to the nature of AS and associating osteoporotic bone pathologies. Despite the clinical importance of initial assessment and accurate diagnosis of traumatic cervical spine injuries, it may be difficult at times because of the site of fracture, overlying shoulders obscuring the lower cervical region in radiographs motion artifact, or other distracting injuries. When a patient has a significant neurologic deficit or severe pain, an intensive examination is indicated to determine whether a fracture is present. Routine CT scans for the AS patient with severe pain or neurologic deficit may be helpful in demonstrating a spine fracture.

In conclusion, multiple spine fractures in AS patients should be considered a devastating injury especially in the elderly population. Our case clearly shows that multiple spine fractures in AS patients occurred in elderly population and have an extremely high mortality and unfavorable outcomes regardless of associated trauma or spinal cord involvement. Appropriate screening strategies and treatment plans will need to be developed to improve outcomes in this devastating injury. Routine entire spine multislice CT scans may be helpful in demonstrating an occult fracture.

## Figures and Tables

**Table 1:** Case review of simultaneous spine fractures in patient with AS.

	Onset	AGE	SEX	Site	ASIA	Treatment	Prognosis
Present study	Fell in the bathtub	82	M	C6/C7 L1	A	Conservative, steroids and Halo traction	Died by pulmonary failure

Osgood et al.	Automobile accident	65	M	C7/T1 T9/T10	B	Conservative, steroids and Halo traction	Died by pulmonary failure

Samartzis et al.	Fell in the bathtub	81	M	C6/C7 T11/T12 L2/L3	A	PSF C3-T3, T11-L5 and Halo traction	Died by pneumonia

## References

[1] Meyer P. (1999). Diffuse idiopathic skeletal hyperostosis in the cervical spine. *Clinical Orthopaedics and Related Research*.

[2] Wang Y.-F., Teng M. M.-H., Chang C.-Y., Wu H.-T., Wang S.-T. (2005). Imaging manifestations of spinal fractures in ankylosing spondylitis. *The American Journal of Neuroradiology*.

[3] Jacobs W. B., Fehlings M. G. (2008). Ankylosing spondylitis and spinal cord injury: origin, incidence, management, and avoidance. *Neurosurgical Focus*.

[4] Shah B. C., Khan M. A. (1987). Review of ankylosing spondylitis. *Comprehensive Therapy*.

[5] Apple D. F., Anson C. (1995). Spinal cord injury occurring in patients with ankylosing spondylitis: a multicenter study. *Orthopedics*.

[6] Broom M. J., Raycroft J. F. (1988). Complications of fractures of the cervical spine in ankylosing spondylitis. *Spine*.

[7] Einsiedel T., Schmelz A., Arand M. (2006). Injuries of the cervical spine in patients with ankylosing spondylitis: experience at two trauma centers. *Journal of Neurosurgery: Spine*.

[8] Hendrix R. W., Melany M., Miller F., Rogers L. F. (1994). Fracture of the spine in patients with ankylosis due to diffuse skeletal hyperostosis: clinical and imaging findings. *American Journal of Roentgenology*.

[9] Hitchon P. W., From A. M., Brenton M. D., Glaser J. A., Torner J. C. (2002). Fractures of the thoracolumbar spine complicating ankylosing spondylitis. *Journal of Neurosurgery*.

[10] Osgood C. P., Abbasy M., Mathews T. (1975). Multiple spine fractures in ankylosing spondylitis. *Journal of Trauma*.

[11] Pérez-López C., Isla A., Gómez Sierra A., Budke M. (2004). Cervical epidural hematoma without fracture in a patient with ankylosing spondylitis. A case report. *Journal of Neurosurgical Sciences*.

[12] Rowed D. W. (1992). Management of cervical spinal cord injury in ankylosing spondylitis: the intervertebral disc as a cause of cord compression. *Journal of Neurosurgery*.

[13] Samartzis D., Liu J. C., Batjer H. H., Loftus C. (2002). Ankylosing spondylitis. *Textbook of Neurological Surgery*.

[14] Surin V. V. (1980). Fractures of the cervical spine in patients with ankylosing spondylitis. *Acta Orthopaedica*.

[15] Trent G., Armstrong G. W. D., O'Neil J. (1988). Thoracolumbar fractures in ankylosing spondylitis. *Clinical Orthopaedics and Related Research*.

[16] Colterjohn N. R., Bednar D. A. (1995). Identifiable risk factors for secondary neurologic deterioration in the cervical spine-injured patient. *Spine*.

[17] Fox M. V., Onofrio B. M., Menezes A. H., Sonntag V. H. (1996). Ankylosing spondylitis. *Principles of Spinal Surgery*.

[18] Grisolia A., Bell R. L., Peltier L. F. (1967). Fractures and dislocations of the spine complicating ankylosing spondylitis. *The Journal of Bone & Joint Surgery—American Volume*.

[19] Olerud C., Andersson S., Svensson B., Bring J. (1999). Cervical spine fractures in the elderly: factors influencing survival in 65 cases. *Acta Orthopaedica Scandinavica*.

[20] Shen F. H., Samartzis D. (2005). Cervical spine fracture in the ankylosing spondylitis patient. *Journal of the American College of Surgeons*.

[21] Finkelstein J. A., Chapman J. R., Mirza S. (1999). Occult vertebral fractures in ankylosing spondylitis. *Spinal Cord*.

[22] Samartzis D., Anderson D. G., Shen F. H. (2005). Multiple and simultaneous spine fractures in ankylosing spondylitis: case report. *Spine*.

[23] Olerud C., Frost A., Bring J. (1996). Spinal fractures in patients with ankylosing spondylitis. *European Spine Journal*.

